# Calibration of cause-specific absolute risk for external validation using each cause-specific hazards model in the presence of competing events

**DOI:** 10.1186/s41512-025-00197-5

**Published:** 2025-10-14

**Authors:** Sarwar I. Mozumder, Sarah Booth, Richard D. Riley, Mark J. Rutherford, Paul C. Lambert

**Affiliations:** 1 Biostatistics Research Group, Department of Population Health Sciences, University of Leicester, Leicester, UK; 2 Statistics and Data Science Innovation Hub, GSK, Biostatistics R&D, London, UK; 3 Department of Applied Health Sciences, School of Health Sciences, College of Medicine and Health, University of Birmingham, Birmingham, UK; 4 National Institute for Health and Care Research (NIHR) Birmingham Biomedical Research Centre, Birmingham, UK; 5 Department of Medical Epidemiology and Biostatistics, Karolinska Institutet, Stockholm, Sweden; 6 Cancer Registry of Norway, Norwegian Institute of Public Health, Oslo, Norway

**Keywords:** Flexible parametric models, Competing risks, Risk prediction, Calibration, Cause-specific hazards

## Abstract

**Background:**

When developing/validating prognostic models, it is typical to assess calibration between predicted and observed risks — either in the development dataset or in an external sample. For competing risks data, correct specification of more than one model may be required to ensure well-calibrated predicted risks for the event of interest. Furthermore, interest may be in the predicted risks of the event of interest, competing events and all-causes. Therefore, calibration must be assessed simultaneously using various measures.

**Methods:**

We focus on the calibration of prediction models for external validation using a cause-specific hazards approach. We propose that miscalibration for cause-specific hazard models be assessed using components specific to each model through the complement of the cause-specific survival alongside the assessment of the calibration of the cause-specific absolute risks. We simulated a range of scenarios to illustrate how to identify which model(s) are mis-specified in an external validation setting. Calibration plots and calibration statistics (calibration slope, calibration-in-the-large) are presented alongside performance measures such as the Brier score and Index of Prediction Accuracy. We use pseudo-observations to calculate observed risks and generate a smooth calibration curve with restricted cubic splines. We fitted flexible parametric survival models to the simulated data to flexibly estimate baseline cause-specific hazards for the prediction of individual cause-specific absolute risks.

**Results:**

Our simulations illustrate that miscalibration due to changes in the baseline cause-specific hazards in external validation data is better identified using components from each cause-specific model. A mis-calibrated model on one cause could lead to poor calibration of the predicted absolute risks for each cause of interest, including the all-cause absolute risk. This is because prediction of a single cause-specific absolute risk is impacted by effects of variables on the cause of interest and competing events.

**Conclusions:**

If accurate predictions for both all-cause and each cause-specific absolute risks are of interest, this is best achieved by developing and validating models via the cause-specific hazards approach. For each cause-specific model, researchers should evaluate calibration plots separately using the complement of the cause-specific survival function to reveal the cause of any miscalibration. However, this also requires careful consideration of dependent censoring which must be sufficiently accounted for.

**Supplementary Information:**

The online version contains supplementary material available at 10.1186/s41512-025-00197-5.

## Background

Calibration is a key component in validating prognostic models as an evaluation of the agreement between the observed risks and the estimated risks from the model(s) at a particular time-point [1, 2]. There are various approaches to assess calibration, either within an internal or external validation setting. This includes presenting a calibration plot with a calibration curve across the whole distribution of predicted risks, which can be supplemented with statistics such as the calibration slope (ideal value of 1), calibration-in-the-large (ideal value of 0) and the observed over expected ratio (ideal value of 1) [3].

Competing events are important to consider when developing or validating prognostic models as they affect the risk of the event of interest and can force a choice in whether to estimate net (sometimes referred to as marginal) measures of risk or report absolute risk estimates. In this paper, we focus on the latter, i.e. predictions of cause-specific absolute risk which provide the real-world risk probability where a patient may experience the other competing events, or causes of death, before the event of interest.

To the author’s knowledge, there have been only a handful of publications that have explicitly addressed approaches to assess calibration in a competing risks setting, namely Gerds et al. (2014), Wolbers et al. (2009, 2014), Austin et al. (2022) and van Geloven et al. (2022), amongst others [1, 4–11]. Most of these papers focus on calibration in the presence of competing risks when only one cause of death is of interest using the Fine and Gray subdistribution hazards approach [12]. However, predicting the risk from each cause of death may be of interest. For example, to determine the impact of cardiotoxic cancer treatment, the patient or clinician may benefit from understanding the risk of dying from cardiovascular disease and other causes alongside their risk of dying from the cancer of interest [13]. Decomposing the total risk of death into cancer and non-cancer outcomes will contribute to a greater understanding of the overall impact [14]. Therefore, we consider competing risks prediction models under the cause-specific hazards approach where the cause-specific absolute risks from each of the competing causes can be estimated. This specific setting has been considered by Hippisley-Cox and Coupland (2017) and van Geloven et al. (2022) [7, 11].

When modelling on the cause-specific hazards scale, a model for each of the competing events (or a combination of competing events which are not of individual interest) and the event of interest are required. This is because each cause-specific model feeds into the prediction of a single cause-specific absolute risk (also referred to as the cause-specific cumulative incidence function). This raises two important questions when developing a prediction model for competing risks data: (1) How well do the models together predict the measure of interest (i.e. cause-specific absolute risk for the events of interest) and (2) how well is each cause-specific model calibrated? Van Geloven et al. (2022) provides a general guide through existing methods and important considerations for the validation of prediction models in the presence of competing risks including calculation of pseudo-observations for calibration, using restricted cubic splines for a smooth calibration curve and reporting appropriate performance measures such as the Brier score [7].

In this paper, we build on the cause-specific hazards approach for prediction modelling in the presence of competing risks and recommend the consideration of the calibration of each cause-specific hazards model using their respective model components for cause-specific predictions using the cause-specific failure function as a supplementary visualisation tool. The observed risks for the corresponding calibration plots are obtained using pseudo-observations which allow estimation of a smooth calibration curve from which calibration statistics such as calibration in the large (CITL) and the calibration slope can be extracted. To appropriately estimate the pseudo-observations for the cause-specific failure function, we use stratification on the prognostic index.

The aim of this paper is to outline the appropriate external validation of prediction models where the estimand of interest is the cause-specific absolute risks for quantifying the risk of each event in the real world in the presence of competing events using the cause-specific hazards approach. We argue that the cause-specific hazards approach is the natural approach to identify miscalibration in both external (and internal) validation settings. The paper begins by providing a brief overview of competing risks approaches with a focus on Royston-Parmar flexible parametric survival models. In the presence of competing risks, dependent censoring should be accounted for when generating pseudo-observations for the 1 minus cause-specific survival predictions which are then used to identify miscalibration in each cause-specific model. We highlight, through a short simulation study, how one can identify miscalibration in each predicted cause-specific absolute risk using the complement of the cause-specific survival function [1]. The dependency between the competing events is simulated through the shared covariates specified for each cause-specific hazards model using the method by Beyersmann et al. [15]. The Brier score, along with the Index of Prediction Accuracy, is presented as useful performance measures along with the calibration slope and CITL statistics to further evaluate miscalibration. Finally, we conclude with a discussion of recommendations, including limitations and areas that require further research.

## Methods

### Evaluating prediction models on the cause-specific hazards scale

There are two main approaches for obtaining predictions of cause-specific absolute risk in the presence of competing risks: Firstly, through combining all cause-specific hazards from separate cause-specific hazard models and, secondly, by modelling directly on a cause-specific absolute risk through the subdistribution hazard function [5, 12, 16].

Further insight into the full impact on prognosis can be obtained by reporting the total absolute risk of the outcome partitioned into component parts due to competing events, that is, not just focussing on the absolute risk of a single event of interest but further evaluating the absolute risk of all competing events given a covariate profile. In this context, it is more natural to focus on the cause-specific hazard modelling approach as it is easier to isolate and model the effect of a covariate on the cause-specific hazard rather than the cause-specific absolute risk (which depends instead on the joint effect of the covariate across all competing events) [6, 17, 18].

#### Cause-specific hazards

Suppose that we have $k=1,\dots , K$ competing events and a nonnegative random variable $T$, with observed survival time, $t$. It follows that the cause-specific hazard function is defined as follows:

1 $$h_5^{cs}\left(t\right)=\underset{\triangle t\rightarrow0}{lim}\frac{Pr\left(t\leq T<t+\triangle t\left|T\geq t,D=k\right.\right)}{\triangle t}$$

which is the rate of dying from a particular cause, $D = k$, at time $t$. The cause-specific absolute risk, ${F}_{k}\left(t\right)$, is derived using all $K$ cause-specific hazard functions as follows:

2 $$F_k\left(t\right)=\int_o^tS\left(u\right)\;h_k^{cs}\left(u\right)du,$$

and,

3 $$S\left(t\right)=\;exp\;\left(-\int_o^t{\textstyle\sum_{j=1}^K}\;h_j^{cs}\;\left(u\right)\;du\right),$$

where $S(t)$ is the all-cause survival function at time $t$.

The cause-specific survival function, ${S}_{k}\left(t\right)=\text{exp}\left(-{\int }_{0}^{t}{h}_{k}^{cs}\left(u\right)du\right)$, is calculated via the nonparametric Kaplan–Meier estimator such that as follows:

$$\begin{array}{c}{\widehat{S}}_{k}^{KM}\left(t\right)= \prod\limits_{j:{t}_{j}\le t}\left(1-\frac{{d}_{kj}}{{n}_{j}}\right),\end{array}$$

where ${t}_{1}<{t}_{2}<\dots <{t}_{j}$ are the observed times to event (for any cause) for each individual $j$ and $\frac{{d}_{kj}}{{n}_{j}}$ is the cause-specific hazard for cause $k$ at time ${t}_{j}$.

In this paper, we strictly utilise the complement of the cause-specific survival function (Eq. 7), which is composed solely from its respective cause-specific hazards model components, as a tool to investigate miscalibration in each cause-specific model on the cause-specific absolute risks (see the “Calibration of the cause-specific absolute risks and each cause-specific hazards model” section). However, due to the presence of competing risks and bias that may occur due to dependent censoring in these predictions, this must be appropriately accounted for. This is discussed further in the “Regression modelling” section.

A nonparametric estimate of the cause-specific absolute risk is obtained using the Aalen–Johansen estimator as follows:

4 $$\widehat F_k^{AJ}\left(t\right)=\sum_{j:t_{j\leq t}}\;\widehat S^{KM}\;\left(t_{j-1}\right)\frac{d_{kj}}{n_j},$$

where $\widehat S^{KM}\;$ is the all-cause Kaplan–Meier survival function [19].

### Regression modelling

Estimating the effect of covariates for cause $k$, ${{\varvec{x}}}_{k}$, on the cause-specific hazards is commonly modelled via the Cox proportional hazards model as follows:

5 $$h_k^{cs}\left(t\left|x_k\right.\right)=h_{0k}^{cs}\left(t\right)exp\left(\beta_k^{cs}\;x_k\right),$$

where ${h}_{0k}^{cs}\left(t\right)$ is the baseline cause-specific hazard function and ${{\varvec{\beta}}}_{k}^{cs}$ is a row vector of regression coefficients for cause $k$, i.e. log-cause-specific hazard ratios. It follows that $\text{exp}({{\varvec{\beta}}}_{k}^{cs}{{\varvec{x}}}_{k})$ gives the effect of the set of covariates ${{\varvec{x}}}_{k}$ on the *rate* of dying from cause $k$. In a cause-specific hazards model, patients with any competing events are censored at the time at which the competing event is observed and assumed to be (conditionally) independently censored. Although the cause-specific survival function can also be obtained solely from the respective cause-specific hazards model, dependent censoring due to the competing event must be accounted for to avoid biased estimation. However, in this paper, the estimand of interest is in the cause-specific absolute risk [20, 21]. Nevertheless, we still utilise estimates of the (complement of the) cause-specific survival function and account for dependent censoring by conditioning for covariates that explain an increased risk in the competing event and the event of interest. Often, the same covariates that impact dependency for one cause usually impact the other. For example, older aged cancer patients will be at higher risk of the competing event of cardiovascular-related deaths and vice versa. Therefore, any covariates that are adjusted for to control for dependent censoring will be included in all of the cause-specific models.

One drawback of the Cox proportional hazards approach is that since the baseline cause-specific hazard, ${h}_{0k}^{cs}\left(t\right)$, is not modelled directly, obtaining absolute risk predictions is more difficult. One approach is to estimate the baseline hazard using a nonparametric approach such as the Breslow estimator, with the potential to also apply smoothing techniques. Estimating the baseline hazard is necessary when developing prediction models in practice and is recommended when performing external validation [22]. Alternatively, we can model the baseline hazard as part of the model using a Royston-Parmar flexible parametric survival model, which uses restricted cubic splines to flexibly model the log-cumulative cause-specific hazard function. A further advantage of the flexible parametric modelling framework is the flexibility to extend to non-proportional cause-specific hazards through inclusion of interaction effects with spline functions for the timescale [23].

Irrespective of the modelling framework, the prediction of absolute risk for a particular outcome event can be obtained by the cause-specific absolute risk. This can be predicted from regression models such that as follows:

6 $${\widehat F}_k\left(t\left|x_k\right.\right)=\int_o^t\widehat S\;\left(t\left|x_k\right.\right)\;\widehat h_k^{cs}\left(u\left|x_k\right.\right)du,$$

where

$$\widehat{S}\left(t|{{\varvec{x}}}_{k}\right)=\prod_{j=1}^{K}\left[ {\widehat{S}}_{j}\left(t | {{\varvec{x}}}_{j}\right)\right]$$

is the all-cause survival function. The complement of all-cause survival function, i.e. the all-cause absolute risk, is as follows:

$$\widehat{F}\left(t|{{\varvec{x}}}_{k}\right)=1-\widehat{S}\left(t|{{\varvec{x}}}_{k}\right)=\sum_{j=1}^{K}{\widehat{F}}_{j}\left(t|{{\varvec{x}}}_{j}\right)$$

In addition, the cause-specific survival function, ${\widehat{S}}_{k}\left(t | {{\varvec{x}}}_{k}\right)$, can be easily obtained analytically from Royston-Parmar flexible parametric survival models without requiring any integration of the cause-specific hazard function since as follows:

$$\begin{array}{c}{S}_{k}^{cs}\left(t | {{\varvec{x}}}_{k}\right)=\mathit{exp}\left(-\mathit{exp}\left( {s}_{k}\left(\mathit{ln}\left(t\right); {\gamma }_{k}, {{\varvec{m}}}_{0k}\right)+ {{\varvec{\beta}}}_{k}^{cs}{{\varvec{x}}}_{k}\right)\right)\end{array}$$

where ${s}_{k}\left(.\right)$ is the restricted cubic spline function that estimates the baseline cumulative hazard function on log time for cause $k$ with $M$ knots.

Finally, we can also obtain model predictions as a function solely of the cause-specific hazards model components. In this paper, we utilise the complement of the cause-specific survival function, $\overline{\widehat{{S}_{k}}}\left(t|{{\varvec{x}}}_{k}\right),$ from a regression model to later evaluate model miscalibration such that as follows:

7 $${\overline{\widehat S}}_k\left(t\left|x_k\right.\right)=1-\widehat{S_k}\left(t\left|x_k\right.\right),$$

An important caveat to the above is that the independent censoring assumption may no longer be plausible, i.e. we may have dependent censoring. Although this is an untestable assumption, the independent censoring assumption becomes more plausible by conditioning on the covariates included in the regression model. However, in reality, we can never test this assumption based on observed data to assess its validity. Therefore, this requires a strong assumption which we might consider to be more plausible based on the more relaxed assumption of conditionally independent censoring to account for any potential dependency in the observed data [24]. The plausibility of this assumption will depend on whether all covariates that measure dependency between competing events are available. In reality, this is unlikely to be captured entirely in the measured covariates available in the data. However, practically speaking, the impact of dependent censoring is likely greatly reduced by conditioning on the available covariate information in the data, provided that the assumption that our prognostic model is reasonable. In this paper, we are primarily interested in only using these cause-specific model components, i.e. complement of the cause-specific survival, to help identify miscalibration on the cause-specific absolute risks. Therefore, since optimal calibration of the complement of the cause-specific survival is not the goal, as long as we reduce dependent censoring as far as possible with what we have available in the data, this should provide enough information in the diagnostic calibration plots based on functions of each cause-specific model components to identify miscalibration on the cause-specific absolute risk.

### Calibration plots

Assessing calibration is key to ensuring appropriate validation of a prediction model. Our work builds on that of Gerds et al. [1], who propose the estimation of calibration curves for competing risk models based on the jack-knife pseudo-observations and the nearest neighbourhood smoother when a single event is of interest [1]. Royston (2014) also proposes the estimation of individual observed event times using pseudo-observations [25]. We extend these ideas to the cause-specific hazards modelling setting and propose that smooth calibration curves are obtained using restricted cubic splines. Importantly, calibration is also evaluated using a function of components from each respective cause-specific hazards model separately to ensure each cause-specific hazards model is well-calibrated.

### Calibration of the cause-specific absolute risks and each cause-specific hazards model

When evaluating calibration of prediction models in the presence of competing risks on the cause-specific hazards scale in external validation data, there are two key issues. Firstly, one must assess whether the cause-specific predicted absolute risks are well calibrated. Secondly, in the case of miscalibration on a cause-specific predicted absolute risk, one must identify which cause-specific hazards model(s) are mis-specified. Since predicted cause-specific risks are composed of parameters from a combination of each individual cause-specific model, if there is miscalibration, it could be any one, and not necessarily all the cause-specific models that are mis-specified. To identify which model is mis-specified, we propose assessing calibration for the 1 minus cause-specific survival estimate (as defined in Eq. 7) in addition to the cause-specific absolute risks. This is proposed as an additional tool for performing calibration checks in external validation data, which is critical for identifying miscalibration on the scale of interest [26].

### Pseudo-observations

In calibration plots, predicted risks (as estimated from the prognostic model) are compared to observed risks. Usually, the observed risks are calculated via nonparametric estimation. For example, the Aalen-Johansen estimator is used for the cause-specific absolute risk, or the complement of the Kaplan–Meier estimator is used for the cause-specific failure function. However, a drawback of these approaches is that predicted and observed risks must be categorised into risk groups, which are usually spread over pre-determined equally spaced, or clinically pre-defined percentiles of the predicted risk distribution. Gerds et al. [1] and Royston (2014) propose estimating observed risks using pseudo-observations which does not require grouping, thus allowing comparisons with individual predicted risks over the entire predicted risk distribution [1, 25].

Pseudo-observations, $\widehat{{\theta }_{i}}$, can be calculated using a jack-knife procedure which replaces the “incomplete” survival data with complete survival data. These are obtained such that as follows:

8 $${\widehat\theta}_{ik}=n\;{\widehat\theta}_k-\left(n-1\right)\;\widehat\theta_k^{-i},$$

where ${\widehat{\theta }}_{k}$ is a suitable nonparametric estimator of $E\left(I(T<t, K=k)\right)$ for cause $k$ with survival time $T$ and ${\widehat{\theta }}_{k}^{-i}$ is the nonparametric estimator calculated on a sample of $n-1$ for cause $k$, which excludes the ${i}^{th}$ individual [27]. When obtaining pseudo-observations for the cause-specific absolute risk, the Aalen-Johansen estimator is used for ${\widehat{\theta }}_{k}$.

#### Calculating observed predictions using the cause-specific Kaplan–Meier estimator in the presence of competing risks/dependent censoring

To fully assess which of the cause-specific hazard models are leading to miscalibration, we propose also calculating calibration curves using the complement of the cause-specific survival function (Eq. 7). This isolates the covariate effects by each cause and reinstates the one-to-one relationship between the covariate effects and the risk measure. The complement of the cause-specific Kaplan–Meier estimate is the natural choice for calculating the pseudo-observations for the observed estimates in Eq. 8. However, in the presence of competing risks, the complement of the cause-specific Kaplan–Meier estimate may not always sufficiently estimate observed 1 minus cause-specific survival probabilities if there was potential dependent censoring between the two competing events. To circumvent this, the predicted 1 minus cause-specific survival probability distribution is categorised into $R$ groups, with values $r=1, 2, \dots , R$, giving a categorical covariate, $Z$. Even though in reality we can never truly test whether the independence censoring assumption is valid, this assumption can be weakened by accounting for some of the dependency by conditioning on this covariate given that the categories sufficiently group patients that have similar risk and the independent censoring assumption is more valid within each risk group. This leads to a more plausible assumption of conditionally independence censoring by accounting for the categorical covariate, $Z$, assuming that the risk groups appropriately capture the censoring distribution [24]. The choice of $R$ depends on the number of observations and the events within each group $r$. However, to minimise bias and to explain as much of the dependent censoring as possible, a sufficient number of groups at equal percentiles across the distribution of the linear predictor must be generated. For example, as long as there are enough events per group, we could create 10 groups defined by each tenth over the predicted risk distribution. In general, too many groups relative to the sample may lead to too few or even no events in one group. Based on all observations, the nonparametric estimator for ${\widehat{\theta }}_{k}$ in Eq. 8 is replaced by the “mixture” Kaplan–Meier estimate as follows:

9 $${\widehat\theta}_k=\widehat S_k^{MKM}\left(t\right)=\sum_{r=1}^Rp_{r,k}\widehat S_{r,k}^{KM}\left(t\right),$$

where ${p}_{r,k}$ is the proportion of individuals with $Z=r$ for cause $k$ and ${\widehat{S}}_{r,k}^{KM}\left(t\right)$ is the Kaplan–Meier estimator for group $r$ and cause $k$ [27]. In practice, we calculate the pseudo-observations for each individual within each group $Z=r$ separately and then apply ${\widehat{S}}_{r,k}^{KM}$ for the nonparametric estimator for ${\widehat{\theta }}_{k}$. Andersen and Pohar Perme (2010) show that this weakens the independence censoring assumption by grouping “similar” patients together through categorisation of the cause-specific linear predictor. Stratifying by this allows us to obtain a reasonably accurate nonparametric estimate of the 1 minus cause-specific survival probability which is used in the calculation of the pseudo-observations [27]. However, the accuracy of the amended “mixture” estimate by grouping patients with similar risk will depend on the assumption that risk groups $r$ cover all shared covariates between the two cause-specific models. In addition, it is imperative to consider the extent to which the dependent censoring assumption has been accounted for in the linear predictor from the development data. This is because the risk groups represent the dependency structure as identified by the linear predictor, and, therefore, this must also sufficiently represent the dependency structure between the event of interest and competing events in the external dataset.

### Smoothing the calibration curve using restricted cubic splines

Gerds et al. [1] propose the nearest neighbour method for smooth curves on the calibration plot which requires selection of an appropriate bandwidth (1). Here, we smooth the calibration curve using restricted cubic splines, however, one may choose alternative smoothers, such as the nearest neighbour or loess method. Details on how to derive the basis functions for the spline function are detailed elsewhere [28].

After calculating ${\widehat{\theta }}_{ik}$ for all individuals, these can be used as the dependent variable in a generalised linear model against the derived spline variables for the predicted risks to produce a smoothed calibration curve with a specified link function. We use a logit link function ensuring the overall calibration curve is constrained between 0 and 1. Here, the pseudo-observations are utilised to calculate an “observed” risk over the full risk distribution to compare with their individual predicted risk without the need for grouping.

Although this foregoes the problem of selecting an appropriate bandwidth as in the case of the loess smoother or nearest neighbour method, one must still choose an appropriate number of degrees of freedom (i.e. knots). This will depend on the complexity in the shape of the calibration curve. An additional consideration is on knot placement. We choose knot locations where the boundary knots are placed at the 5th and 95th percentiles of the distribution of predicted risk [29]. However, we can choose to place the boundary knots at more extreme percentiles with larger samples.

To supplement the presentation of the curve, we further propose to plot the marginal observed risks versus predicted risks for groups as defined between chosen percentiles over the distribution of predicted risk (in a similar way that risk groups are added to traditional calibration plots). For example, when calibration curves are presented over grouped predictions, the choice of 10 risk groups is commonly used (defined by tenths of the predicted risk distribution).

### Performance measures

The C-index, or concordance index, is commonly reported alongside calibration plots [5, 30]. Such measures evaluate discrimination which can aid in identifying suboptimal predictive accuracy alongside calibration. It is also useful to show the calibration together with summaries to aid interpretation, such as the integrated calibration index or the maximal absolute difference between observed and predicted probabilities of the outcome, i.e. ${E}_{max}$ [6, 29]. In this paper, to evaluate model performance with regard to both calibration and discrimination, i.e. overall performance, we focus on the Brier score and the index of prediction accuracy (IPA) [1, 30]. The Brier score is also described for the 1 minus cause-specific survival probability using a model for the censoring distribution using inverse probability censoring weights (IPCW); however, this can also be obtained using a pseudo-observations approach as described by Cortese et al. [31] [31]. We demonstrate here using the IPCW approach to illustrate another method for accounting for dependent censoring. In addition to the Brier score, we also utilise calibration statistics, i.e. the calibration slope and calibration-in-the-large measures which can easily be obtained at a specific time-point using a similar regression model fitted for the smoothed calibration curve in the “Smoothing the calibration curve using restricted cubic splines” section, but instead use the fitted linear predictor as the independent variable.

#### Brier score

The Brier score for a competing risk survival model can be obtained using inverse probability censoring weights [32, 33]. The Brier score for the ${k}^{th}$ cause-specific absolute risk is as follows:

10 $$\begin{array}{c}{\widehat B}_k\left(t\right)=\frac1N\sum\limits_{i=1}^N\left[\frac{\sum_{j=1}^KI\left(T_i\leq t,K_i=j\right)\left(I\left(T_i\leq t,K_i=k\right)-{\widehat F}_j\left(t\right|{\boldsymbol x}_{ij})\right)^2}{G_i\left(T_i\right)}+\frac{\sum_{j=1}^KI\left(T_i>t\right)\left(I\left(T_i\leq t,K_i=k\right)-{\widehat F}_j\left(t\right|{\boldsymbol x}_{ij})\right)^2}{G_i\left(t\right)}\right],\end{array}$$

where ${G}_{i}\left(t\right)$ is the censoring distribution for the ${i}^{th}$ individual, i.e. probability of not being censored by time $t$. The Brier score for cause $k$ (in the presence of competing risks) ranges from 0 (perfect model) to 0.25 (model with no prognostic variables) where the absolute risk for the event of interest at time $t$ is 50%. The upper value for a model with no prognostic factors will vary dependent on the proportion of events.

Similarly, the Brier score at time $t$ for the complement of the cause-specific survival function can be calculated as follows:

11 $$\begin{array}{c}\widehat B_k^{model}\left(t\right)=\frac1N\sum\limits_{i=1}^N\left[\frac{I\left(T_i\leq t,K_i=k\right)\left(I\left(T_i\leq t,K_i=k\right)-\overline{{\widehat S}_k}\left(t\right|{\boldsymbol x}_{ik})\right)^2}{{G_i\left(T_i\right)G}_{ki}\left(T_i\right)}+\frac{I\left(T_i>t\right)\left(I\left(T_i\leq t,K_i=k\right)-\overline{{\widehat S}_k}\left(t\right|{\boldsymbol x}_{ik})\right)^2}{G_i\left(t\right)G_{ki}\left(t\right)}\right],\end{array}$$

where $\overline{{\widehat{\text{S}} }_{k}}$ denotes the complement (i.e. 1 minus) of the cause-specific survival probabilities predicted from the respective cause-specific hazards model. For the above, additional considerations are required on the model for the censoring distribution for each cause, ${G}_{ki}\left(t\right)$, due to dependent censoring of the competing event. We use IPCW for the competing event to account for the dependency. An approach similar to Lambert et al. (2017) is adopted, which fits a cause-specific flexible parametric model with time-dependent effects to calculate the weights to account for individuals who experience the competing event. These are then applied to those who do not experience the competing event [17]. Similarly, as highlighted in the “Calculating observed predictions using the cause-specific Kaplan–Meier estimator in the presence of competing risks/dependent censoring” section, the model for the censoring distribution is assumed to sufficiently capture the covariates that explain dependency between the competing events. To account for the dependency as much as possible that results in the more plausible conditionally independent censoring assumption, a relatively complex censoring model (with time-dependent effects) that includes the union of covariates that are present in the analysis model for each cause is used. The censoring model itself of course must be sensible enough which is informed by the disease area and research question. However, the validity of the censoring model can never be confirmed in practice.

#### Index of Prediction Accuracy

Interpretation of the Brier score depends on ${F}_{k}(t)$. For example, if ${F}_{k}(t)$ is lower, the scale of the Brier score is also smaller which means that even though generally a lower Brier score is better, this depends on the cause-specific event risk observed in the data. Therefore, due to this ${F}_{k}(t)$ dependent sliding scale on the interpretation of the Brier score, the IPA has been proposed [34]. The IPA measure rescales the Brier score by comparing the prediction model’s Brier score to some benchmark model. This benchmark model can be any null model with no covariates. Therefore, it follows that the IPA can be interpreted in a similar way to the ${R}^{2}$ measure such that 1 indicates a perfect model, 0 represents a “useless” model and any value less than 0 identifies harmful models. The IPA can be calculated as follows:

12 $$\begin{array}{c}IPA=1-\frac{{\widehat B}_k\left(t\right)}{{\widehat B}_{k,null}\left(t\right)},\end{array}$$

where ${\widehat{B}}_{k,null}\left(t\right)$ is the Brier score for the null model fitted with no covariates.

##### Choosing the null model

A common choice for the null model is a nonparametric estimator. For example, for the cause-specific absolute risk, the Aalen-Johansen estimator is usually used [34]. On the other hand, for 1 minus cause-specific survival probabilities, one must suitably account for dependent censoring in the null model.

To calculate the null model’s Brier score for the 1 minus cause-specific survival probability, we model the censoring distribution for each cause as detailed in the “Brier score” section and apply this to the nonparametric estimate of the Kaplan–Meier function.

### Simulation design

A simulation study was undertaken to demonstrate the benefits of considering predictions constructed solely from components from the respective cause-specific hazards model when evaluating miscalibration in a competing risks setting. We aim to demonstrate the merits of this approach in external model validation (i.e. if the baseline risk of one cause is different in a new setting). To achieve this, we simulate large datasets under competing risks as the derivation data and simulate a validation sample with three different baseline cause-specific absolute risks.

The competing risks data is simulated using a cause-specific hazards driven approach as described by Beyersmann et al. (2009) [15]. Simulation details and simulated baseline cause-specific absolute risk (or cumulative incidence functions) and hazard functions are provided in. In summary, a proportional effect on the cause-specific hazards are simulated between covariates,$x$, with administrative censoring at $t=10$ years. Continuous covariates, ${x}_{k1},$ ${x}_{k2}$ and ${x}_{k3},$ are also simulated with a non-linear functional form for${x}_{k1}$, as well as a categorical variable,${x}_{k4}$, with four levels.

The derivation, i.e. model building data, was generated with 50,000 observations. A large dataset was simulated to minimise the impact of sampling variation. The correctly specified data-generating model used to generate the derivation data will be compared with the calibration plots generated in the scenarios that follow. Specifically, in the “A correctly specified prediction model (using DGM) in derivation data” section, the DGM is evaluated. Independent validation datasets were also simulated with 50,000 observations, which had the following:

1. A higher baseline cause-specific absolute risk for the competing event compared to the derivation data. An applied example where this may occur could be when applying a developed prediction model to an external population with higher comorbidities. This is shown by the red line in Fig. A2. This scenario is explored in the “Change in baseline cause-specific hazard for competing event (cause 2) in validation data” section. where the DGM for the derivation model is fitted in the validation data with a higher baseline cause-specific absolute risk.
2. A change in the baseline cause-specific hazard for cause 1 compared to the derivation data, which leads to a higher baseline cause-specific absolute risk for cause 1 by time 10. This is shown by the orange line in Fig. A2, and this scenario is illustrated in the “Change in baseline cause-specific hazard for cause 1 in validation data” section.
3. Both 1 and 2 in the validation data compared to the derivation data which is explored in the “Change in baseline cause-specific hazard for cause 1 and cause 2 in validation data” section. This scenario leads to almost the same predicted baseline absolute risk for cause 1 by time 10 as illustrated by the green line in Fig. A2.

## Results

### A correctly specified prediction model (using DGM) in derivation data

**Fig. 1:**
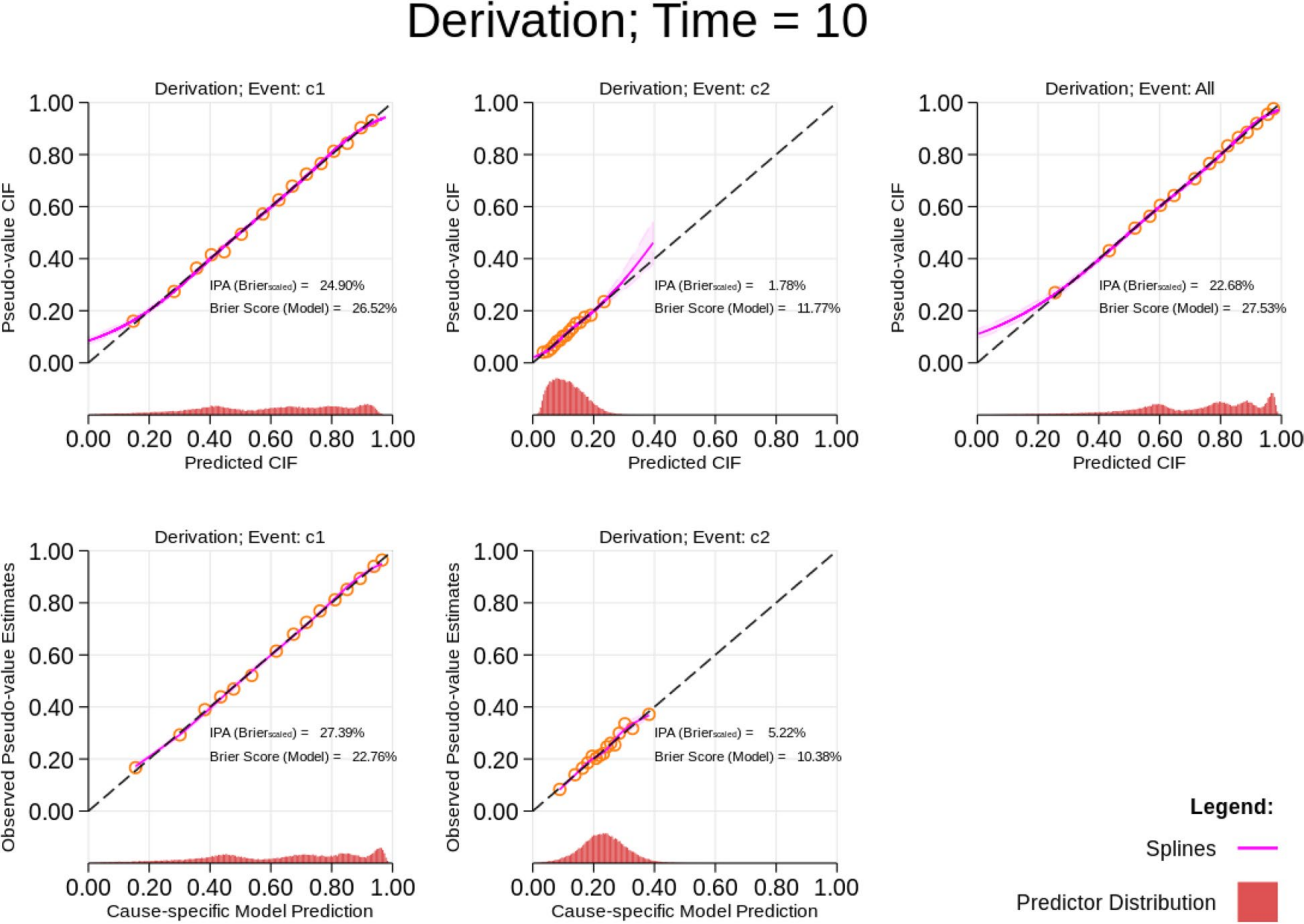
Calibration plots for data-generating model on derivation data at time *T* = 10. Dashed line is a reference line representing perfect calibration. Pink line shows the smoothed calibration curve estimated using pseudo-observations within a logistic regression model. Orange dots show the grouped risks over the predictor distribution. The cumulative incidence function (CIF) refers to the absolute risks

Figure 1 illustrates calibration plots at 10 years for each cause-specific model prediction and absolute risk when the data-generating model from the “Simulation design” section is fitted to the simulated data. As expected, this shows almost perfect calibration. This can be seen by the fact that the regression line (pink line) fitted to the pseudo-observations lies very close to the diagonal line of agreement between the observed and predicted absolute risk and 1 minus cause-specific survival probability estimates. The orange circles represent the scatter of the model predictions and pseudo-observations averaged within 15 risk groups. These risk groups are defined between equal percentiles across the predictor distribution as represented by the histogram at the bottom of the figure. From Fig. 1, we observe that the predicted absolute risks for cause 1 are spread quite well from 0 to 1, with a larger number of observations with a high predicted absolute risk (> 0.8). The predicted absolute risks for cause 2 are mostly between 0 and 0.2. The predicted cause-specific model probabilities for cause 1 and cause 2 are slightly higher than the corresponding absolute risk estimates. Evidently, the smoothed curve deviates from the diagonal line of agreement for the absolute risks. This may occur due to the number of cause-specific events and censoring at the tail for this specific time point, and so one may choose to only plot the smoothed curve up to the boundary knots. Alternatively, one can also attempt to improve the smoothed curves at the tails by changing the location of knots at the boundary. However, we would recommend using default locations as suggested in Harrell [29] to avoid fixing knot locations that are overly dependent on the derivation data [29].

### Change in baseline cause-specific hazard for competing event (cause 2) in validation data

**Fig. 2:**
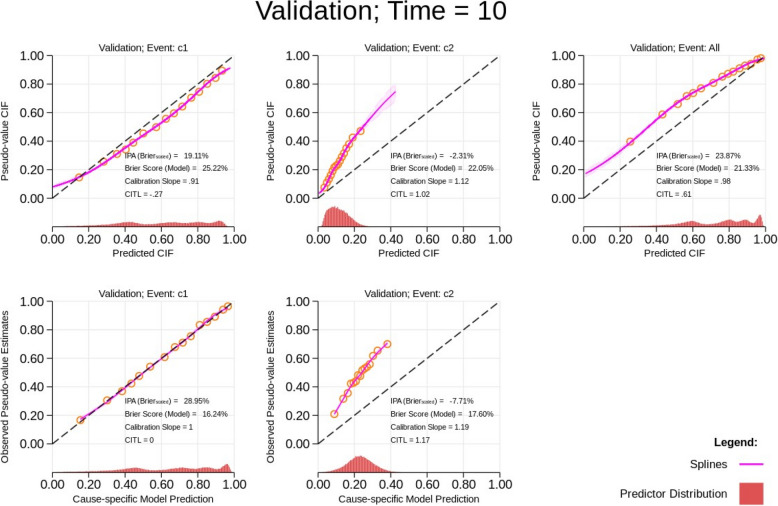
Calibration plots for predictions obtained from a correctly specified model for both causes in an independent validation dataset at time *T* = 10 with a higher baseline hazard for cause 2. The cumulative incidence function (CIF) refers to the absolute risks. CITL, calibration-in-the-large; IPA, index of prediction accuracy

Figure 2 illustrates the calibration plot for the external validation scenario when the absolute risk of cause 2 has substantially increased in the external sample. In this case, the cause-specific absolute risks for each cause show miscalibration (top row, Fig. 2). This is also illustrated by the calibration slope and calibration-in-the-large statistics. It is only by inspecting the cause-specific model predictions where the model that is not performing well can be identified. The calibration curves for the cause-specific model predictions indicate that the cause 2 model does not fit the independent validation data well. This informs which model will require recalibration as this may not always need to be performed for both models. Following this visual evaluation on the extent of miscalibration in the independent validation data at a specific time-point, the researcher can identify which cause-specific model to re-estimate. Even though both cause-specific models can be re-estimated on the independent validation data to see which coefficients change, using the calibration curves using components that correspond to each cause-specific model allows us to further visualise the extent of the change in baseline cause-specific hazards in a more holistic manner. Therefore, we recommend this approach as a useful informative step before diving into the re-estimation of the model(s). The merit of especially reporting the calibration statistics (slope and calibration-in-the-large) is further illustrated in the sections “Change in baseline cause-specific hazard for cause 1 in validation data” and “Change in baseline cause-specific hazard for cause 1 and cause 2 in validation data”.

### Change in baseline cause-specific hazard for cause 1 in validation data

**Fig. 3:**
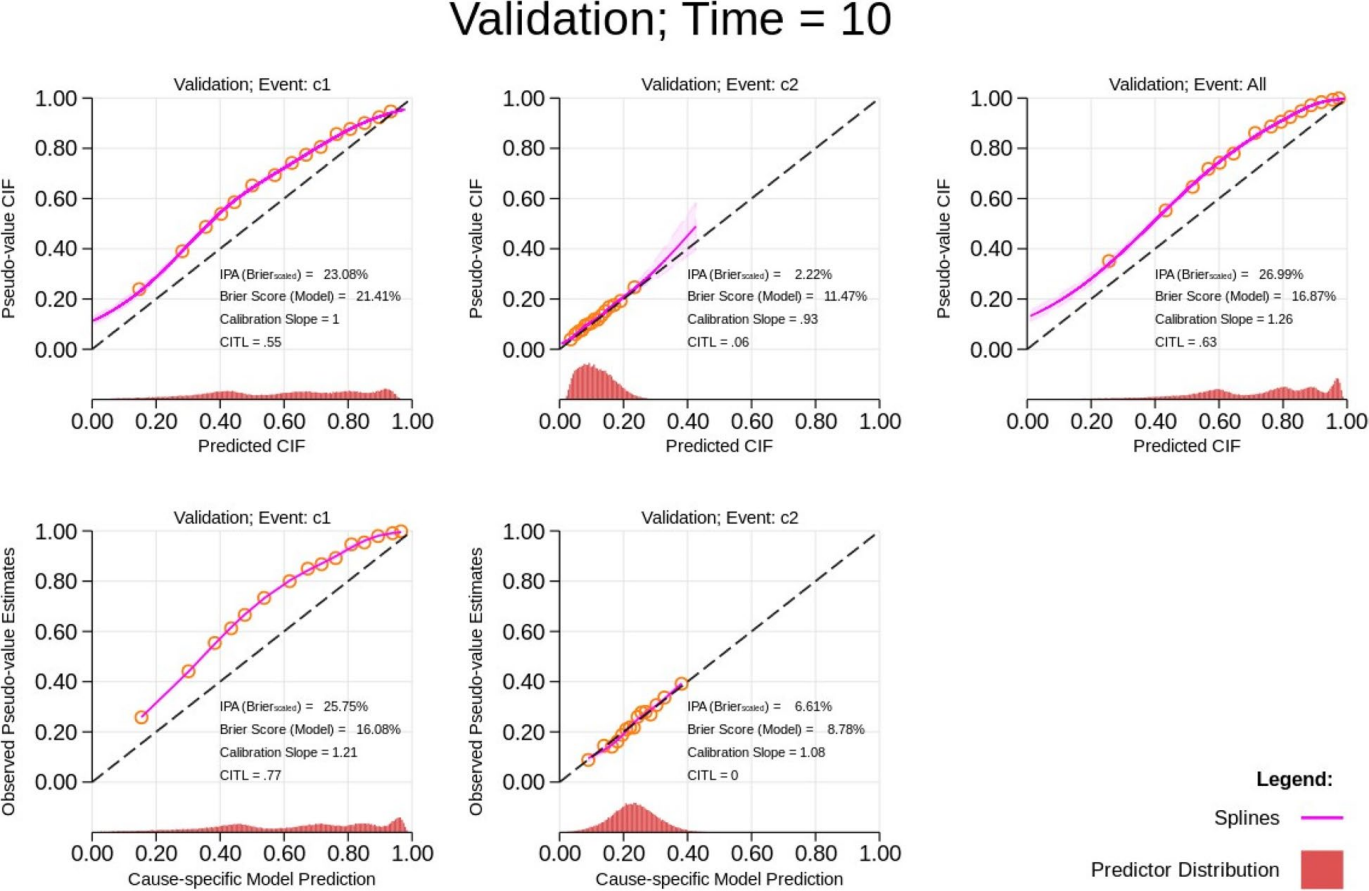
Calibration plots for predictions obtained from a correctly specified model for both causes in an independent validation dataset at time *T* = 10 with a change in the baseline hazard for cause 1 (see orange line in Fig. A2). The cumulative incidence function (CIF) refers to the absolute risks. CITL, calibration-in-the-large; IPA, index of prediction accuracy

Figure 3 presents the scenario when the baseline cause-specific hazard for cause 1 changes in the validation dataset. When we compare the IPA for the cause-specific absolute risks in the calibration plot for the validation dataset in Fig. 3 to those that are presented in Fig. 1, we see little difference between them. In addition, it is important to note that although the calibration slope is equal to 1 for the absolute risk for cause 1, the calibration-in-the-large statistic is not close to 0 (i.e. 0.55). This emphasises the importance of supplementing the calibration slope statistic with calibration-in-the-large when evaluating miscalibration. This is also recommended by Riley et al. [3] in Sect. 3.4.2, Box 3.2, of the book since the magnitude of the baseline cause-specific hazard may not be appropriate even when the calibration slope is 1 as shown in this scenario [3]. Similarly, by observing the calibration statistics and plot for the cause-specific model predictions solely derived using its respective components, we correctly identify miscalibration in the model for cause 1 in the independent validation dataset, since the statistics for the calibration slope and CITL for cause 2 are sufficiently close to 1 and 0, respectively.

### Change in baseline cause-specific hazard for cause 1 and cause 2 in validation data

**Fig. 4:**
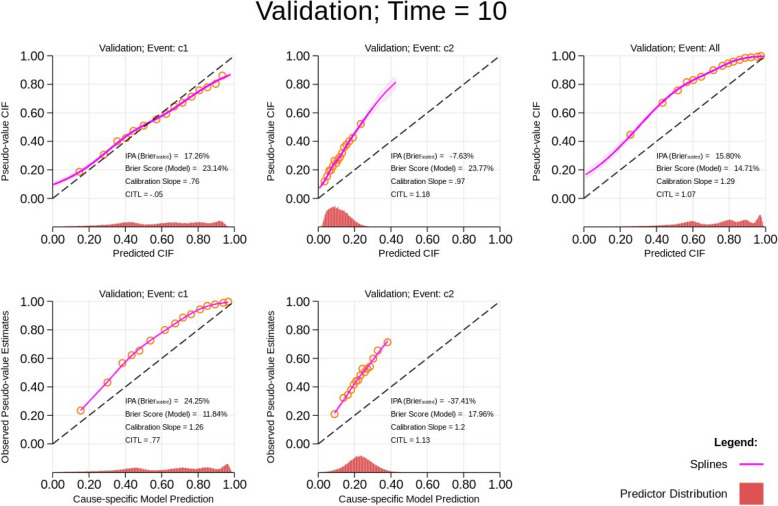
Calibration plots for predictions obtained from a correctly specified model for both causes in an independent validation dataset at time *T* = 10 with a change in the baseline hazard for cause 1 and cause 2 (see green line in Fig. A2). The cumulative incidence function (CIF) refers to the absolute risks. CITL, calibration-in-the-large; IPA, index of prediction accuracy

Finally, in this scenario, in Fig. 4, when we see changes in the baseline hazard for both causes, visually, the calibration looks good in the calibration plot for the absolute risk for cause 1. However, the calibration slope is not close to 1, suggesting some miscalibration. Similarly, for the absolute risk for cause 2, although the calibration slope is close to 1, miscalibration is evident from the calibration-in-the-large measure (1.18). In this case, by evaluating calibration using 1 minus the cause-specific survival function, we can observe that both cause-specific models are miscalibrated with a calibration slope for both causes of more than 1, indicating narrow predictions. The IPA for the cause-specific model predictions in cause 2 also suggests a poorly fitting model compared to the null cause-specific model. Since these scenarios are correctly specified with respect to the covariates included in the models, the miscalibration points to an incorrect magnitude in the cause-specific baseline hazards.

## Discussion

When assessing calibration in the external validation of risk prediction models in the presence of competing risks, we propose that researchers also perform calibration checks using the complement of the cause-specific survival function. Models on the cause-specific hazards scale for the event of interest and competing events are therefore also recommended, where calibration is assessed for both absolute risks (i.e. cause-specific cumulative incidence functions) and cause-specific survival. Although the paper focuses on the application within the Royston-Parmar flexible parametric survival modelling framework, the methods are generalizable to other parametric and semi-parametric models such as cause-specific Cox proportional hazard models. The Brier score and IPA are presented as useful performance measures when evaluating in an external dataset for validation. These measures can be supplemented with alternative measures of calibration and discrimination. In our simulations, we present the calibration slope and calibration-in-the-large statistics that offer an easy to interpret supplement to the visual inspection of calibration plots. For implementation of methods discussed in this paper, a Stata command calplotcr has been written, which is available at https://github.com/sarwarislam/cal-competing-risks. The command is written as an extension of the stbrier and pmcalplot commands [35, 36].

The most notable contributions for developing prediction models in the presence of competing risks mainly focus on subdistribution hazards models, i.e. the Fine and Gray model, and only discuss evaluating calibration on one cause-specific absolute risk of interest [1, 4, 5]. Our work extends ideas by Gerds et al. [1] and Royston (2014) (1, 25). However, rather than modelling and assessing calibration on a single cause of interest in the presence of competing risks, we provide guidance on how to evaluate models on each individual cause and the all-cause predictions using cause-specific hazard models. Furthermore, to obtain a smoothed calibration curve over the individual cause-specific model predictions and absolute risk predictions from the model, pseudo-observations and restricted cubic splines are used. However, using any sensible smoother should lead to similar conclusions. This is consistent with recommendations within an article by van Geloven et al. (2022) which was published at the time of writing this paper [7]. An important element of obtaining pseudo-observations as an estimate of the complement of the cause-specific Kaplan–Meier estimator is to account for dependent censoring as discussed in the “Calculating observed predictions using the cause-specific Kaplan–Meier estimator in the presence of competing risks/dependent censoring” section. Failure to do so may result in biased estimation and requires careful consideration [27]. A key contribution of this paper is the details on how to make accurate comparisons between observed cause-specific predictions as calculated by pseudo-observations and cause-specific model predictions.

A further significant contribution in addition to Gerds et al.’s [1] work is the argument that to build a suitably calibrated competing risks prediction model, we must ensure appropriate calibration on each cause-specific hazards model as well as absolute risks. This is particularly important for cause-specific hazard models, since miscalibration on a cause-specific absolute risk can be more easily identified by evaluating miscalibration of each cause-specific model component. This is because a cause-specific absolute risk is composed of parameters from each cause-specific hazards model. Therefore, one miscalibrated cause-specific model may impact all cause-specific absolute risks and all-cause absolute risk. Although we outline some proposals on evaluating cause-specific prediction models in the presence of competing risks using calibration plots, there are various points not covered in this paper, such as, discrimination-based measures and the utility of the proposed measures for internal validation/model development in comparison to traditional model checking measures. However, in the supplementary material B, we present some simulations on how these methods can be used for internal validation. Traditional model fitting scores on the cause-specific hazards scale should be used in supplement, such as residual checking and using model selection criteria such as AIC and BIC. The main difference from our proposed approach and these traditional model-checking measures is that the latter ensures the model is correctly specified on average over the entire follow-up time. On the other hand, we assess calibration using the cause-specific hazards model predictions to ensure correct model specification at a specific time-point of interest. For internal validation, since the cause-specific absolute risks are likely to be well calibrated on average, sometimes this makes it difficult to identify miscalibration and model misspecification visually (see, for example, Fig. B2 in supplementary material). In such cases, additional overall prediction measures, such as the Brier score and IPA, can be used to supplement the assessment of miscalibration, including other calibration statistics and discrimination measures.

In this paper, to assess miscalibration in external data in each cause-specific model, we propose utilising the cause-specific probabilities derived solely from the components for each cause. This does not circumvent the need to account for dependent censoring due to the presence of competing events and the censoring model used in the “Brier score” section. To calculate the Brier score requires careful thought, as a reviewer has rightly highlighted. The dependency between the competing events may be different for the validation and development data, and the dependency must be sufficiently accounted for to compare the observed and estimated probabilities derived from the cause-specific model. In practice, whether or not the censoring model is valid (in either the development or validation data) can never be verified; however, we must ensure the dependent censoring is modelled as much as possible with the data available. The censoring model is primarily informed by the disease area and research question in collaboration with clinical stakeholders. In reality, we do not need to fully satisfy this assumption (in fact, we can never do this or verify we have done this), but we must attempt to address this as far as possible to the best of our knowledge. To strengthen the censoring model, we may also model for time-dependent covariates (if available) that contain information on how a patient’s risk, or probability of being censored (due to the competing event), may change over time. One suggestion could be to perform sensitivity analyses by visualising the change in calibration slope for different censoring models. Finally, it is important to consider that the dependency structure (the censoring model) may change in the external dataset. Again, a similar sensitivity analysis can be performed; however, it is unlikely that the dependency structure will change or be too dissimilar in the external validation data as it was within the derivation dataset. Alternatively, as we have proposed for the pseudo-value approach to obtain observed 1 minus cause-specific survival in the calibration plot, we can use stratification by the prognostic index to account for the dependent censoring due to the competing event.

An important element when validating a prediction model is in model selection and determining appropriate model shrinkage. Previously, much of the literature in this area has focussed mainly on models directly on the absolute risks, e.g. the Fine and Gray subdistribution hazards model, rather than through the cause-specific hazards [37, 38]. Model selection methods for the former must consider covariates that impact either the competing events or the event of interest even if a single model for the event of interest is developed. Careful thought should be given to the impact of external validation of a subdistribution hazard model. It may not be simple to appropriately recalibrate in scenarios when the baseline absolute risk of one of the causes is altered in a new setting. Fitting separate cause-specific models circumvents such issues but means that we lose the direct one-to-one relationship between the parameters and the cause-specific absolute risks.

Finally, when evaluating and updating an existing model to a new dataset for external validation, application of the methods outlined in this paper relies on the sufficient reporting of the clinical prediction model. This includes reporting all cause-specific hazards model coefficients so that these can be easily extracted for the refitting of the cause-specific baseline hazard in the new data. Therefore, we strongly recommend that researchers consider reporting the cause-specific baseline hazards at various time-points up to and including the time-point of interest for prediction. Although reporting guidelines, such as TRIPOD, require sufficient transparency of developed prediction models, item 15 only recommends that authors of prediction models present all regression coefficients and baseline survival (or hazard) at a given time-point [39]. However, when considering cause-specific hazard models and competing risks, this recommendation should be extended to present all coefficients in each cause-specific hazards model and baseline hazard at multiple time-points up to the time-point of interest. As one reviewer correctly highlighted, it is vital that we try to appropriately account for covariates that may drive dependent censoring when interest is in cause-specific survival measures (be that through appropriately modelling covariate effects for the cause-specific model-based estimates or appropriately stratifying when constructing the pseudo-observations for the complement of the cause-specific survival). Whether the dependency structure has been properly accounted for is untestable, and this itself may not always be observable in the data. Evidently, assessing the impact of the violation of this assumption requires further work and is a key topic for future research, particularly if there are differential drivers in an external validation setting. However, we would like to emphasise which does not rely on the independent censoring assumption. The complement of the cause-specific survival measure, which is a function of the respective cause-specific model components, is used solely as an evaluation tool to identify which model may be leading to miscalibration for the cause-specific absolute risks. Ultimately, we propose reporting of the cause-specific absolute risk, and this is the estimand we are focussed on ensuring that is well-calibrated. As long as we attempt to account for any dependent censoring that might be present with the covariates collected and available in the data, our approach enables us to identify more clearly which cause-specific model is leading to miscalibration on the cause-specific absolute risk.

## Conclusion

To conclude, we present some additional tools for evaluating calibration and accuracy of prediction models for competing risks using cause-specific hazards models. Through various scenarios, we demonstrate how calibration plots, statistics and performance measures can be utilised for cause-specific absolute risks and cause-specific predictions associated with the cause-specific hazards model to identify model mis-specification when performing external validation. Finally, we outline appropriate methods to ensure accurate estimation of observed cause-specific model probabilities from cause-specific hazard models in the presence of competing risks for use in calibration plots and for the calculation of the Brier score for the null model.

## Supplementary Information

Appendix A: Simulation Design: Figure A1: Simulated cause-specific hazard ratios (CHR) for each variable, assuming baseline (0) values for all other variables (top row for cause 1, bottom row for cause 2). Table A1: Cause-specific hazard ratios for variable. Figure A2: Simulated baseline cause-specific hazards and cumulative incidence functions where all variables have baseline values of 0 with an additional independent validation dataset with a different baseline hazard for cause 1 (orange) and a higher incidence of cause 2 (red).
Appendix B: Demonstration of Proposed Method for Internal Validation. Figure B1: Calibration plots on the derivation data for predictions obtained from a correctly specified model for cause 2, and a mis-specified model with the incorrect functional form for for cause 1 at time T = 10. The cumulative incidence function (CIF) refers to the absolute risks. Figure B2: Calibration plots on the derivation data for predictions obtained from a correctly specified model for cause 2, and a mis-specified model with a variable not included for cause 1 at time T = 10. The cumulative incidence function (CIF) refers to the absolute risks.

## Data Availability

All data generated or analysed during this study are included in this published article within the appendix.
