# Supplementary material for: Calibration of cause-specific absolute risk for external validation using each cause-specific hazards model in the presence of competing events
Source: Diagn Progn Res. 2025 Oct 14;9:23. doi: 10.1186/s41512-025-00197-5 (PMC12519608; doi:10.1186/s41512-025-00197-5)
Supplement: Supplementary file 1 — Appendix A: Simulation Design: Figure A1: Simulated cause-specific hazard ratios (CHR) for each variable, assuming baseline (0) values for all other variables (top row for cause 1, bottom row for cause 2). Table A1: Cause-specific hazard ratios for variable. Figure A2: Simulated baseline cause-specific hazards and cumulative incidence functions where all variables have baseline values of 0 with an additional independent validation dataset with a different baseline hazard for cause 1 (orange) and a higher incidence of cause 2 (red). Appendix B: Demonstration of Proposed Method for Internal Validation. Figure B1: Calibration plots on the derivation data for predictions obtained from a correctly specified model for cause 2, and a mis-specified model with the incorrect functional form for for cause 1 at time T = 10. The cumulative incidence function (CIF) refers to the absolute risks. Figure B2: Calibration plots on the derivation data for predictions obtained from a correctly specified model for cause 2, and a mis-specified model with a variable not included for cause 1 at time T = 10. The cumulative incidence function (CIF) refers to the absolute risks. [file 41512_2025_197_MOESM1_ESM.docx]

# Appendix A: Simulation Design

Cause-specific hazard functions were simulated for 2 events, each of which were generated from a two parameter mixture Weibull distribution, which assumes a proportional effect on the cause-specific hazards scale between covariates, $\boldsymbol{x}$. Simulating from such a distribution allows for a complex function with one or more turning points which is often synonymous with the behaviour of hazard functions in real-world data (1).

The cause-specific hazard function for event $k$ was simulated such that,

$$h_{k}^{\text{cs }}\left( t \right)=\frac{\lambda_{k1}\gamma_{k1}p_{k}\exp\left( -\lambda_{k1}t^{\gamma_{k1}} \right)+\lambda_{k2}\gamma_{k2}t^{\gamma_{k1}-1}\left( 1-p_{k} \right)\exp\left( -\lambda_{k2}t^{\gamma_{k2}} \right)}{p_{k}\exp\left( -\lambda_{k1}t^{\gamma_{k1}} \right)+\left( 1-p_{k} \right)\exp\left( -\lambda_{k2}t^{\gamma_{k2}} \right)}exp\left( \boldsymbol{x}_{\boldsymbol{k}}\boldsymbol{\beta}_{k} \right)$$

where $\lambda_{k}$, $\gamma_{k}$ and $p_{k}$ are the shape, scale and mixture parameters for event $k$. Survival times were then simulated from the all-cause hazard function, $h_{1}^{cs}\left( t \right)+h_{2}^{cs}(t)$ with administrative censoring at 10 years.

Continuous covariates, $x_{k1}$, $x_{k2}$ and $x_{k3}$ for each cause $k$ were drawn independently from a normal distribution with means 0 and standard deviations 1, 1 and 0.3 respectively. A categorical variable, $x_{k4}$, with 4 groups was also simulated from a single uniform distribution $z \sim Uniform(0,1)$, where,

$$x_{k4}= \left\{ \begin{aligned} 0 if z<0.25 \\ 1 if 0.25\leq z<0.50 \\ 2 if 0.50\leq z<0.75 \\ 3 if z\geq0.75 \end{aligned} \right.$$

Each cause-specific hazard ratio was assumed to have a proportional effect and a quadratic term was included for $x_{k1}$. The simulated log-cause-specific hazard ratios are presented in Figure A1 for covariates $x_{k1}$, $x_{k2}$ and $x_{k3}$ and Table A1 for $x_{k4}$. Note that these are deliberately intended as extreme scenarios to clearly illustrate the methods.


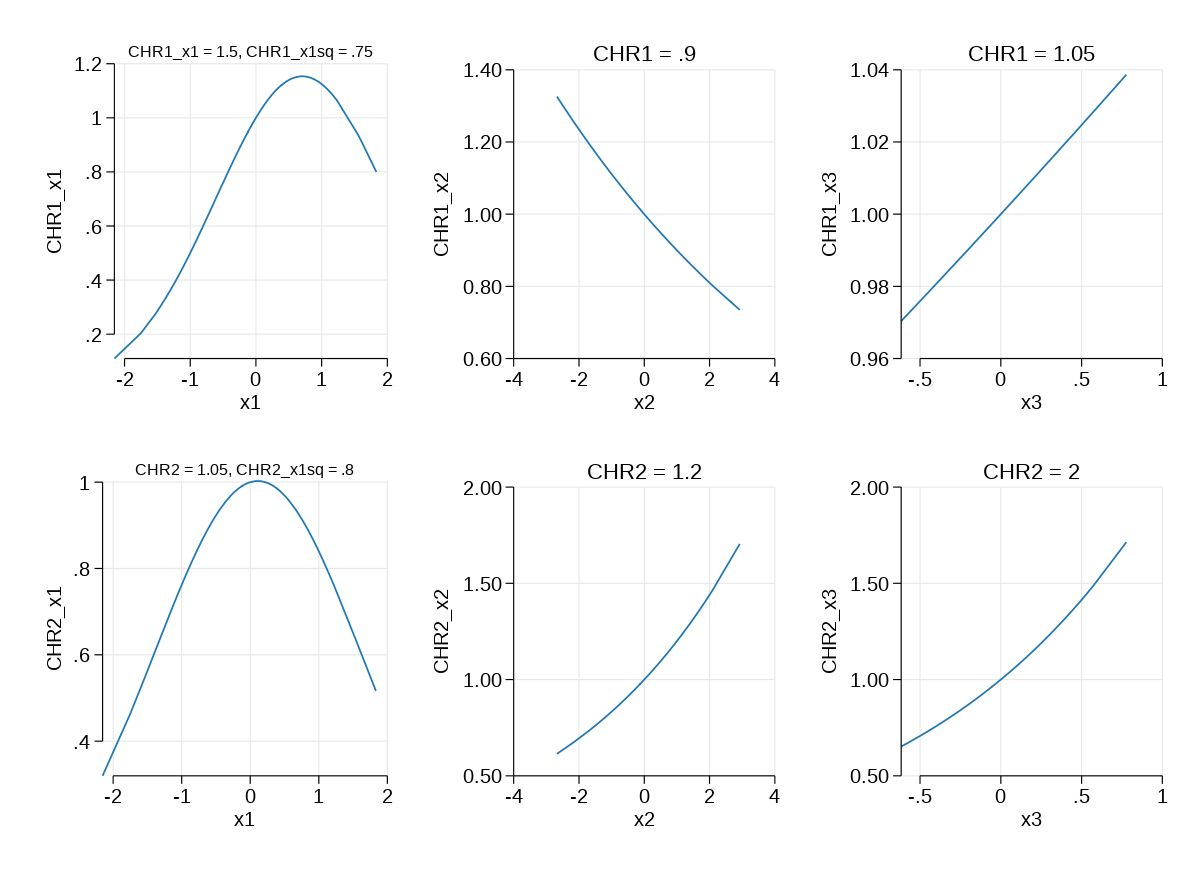


Figure A1: Simulated cause-specific hazard ratios (CHR) for each variable, assuming baseline (0) values for all other variables (top row for cause 1, bottom row for cause 2)

Table A1: Cause-specific hazard ratios for variable $x_{k4}$

| **Group (**$\boldsymbol{x}_{\boldsymbol{k}\boldsymbol{4}}$**)** | **Hazard ratio for cause 1** | **Hazard ratio for cause 2** |
| --- | --- | --- |
| 0 | 1 | 1 |
| 1 | 2 | 1.2 |
| 2 | 3 | 1 |
| 3 | 5 | 1 |

The simulated baseline cause-specific hazard functions and baseline cause-specific cumulative incidence functions are presented in Figure A2 (blue lines). These will vary by the simulated covariates, but since these are presented at baseline, the cause-specific hazards and incidence functions are illustrated at the mean values of covariates, $x_{k1}$, $x_{k2}$ and $x_{k3}$ and at $x_{k4}=0$.


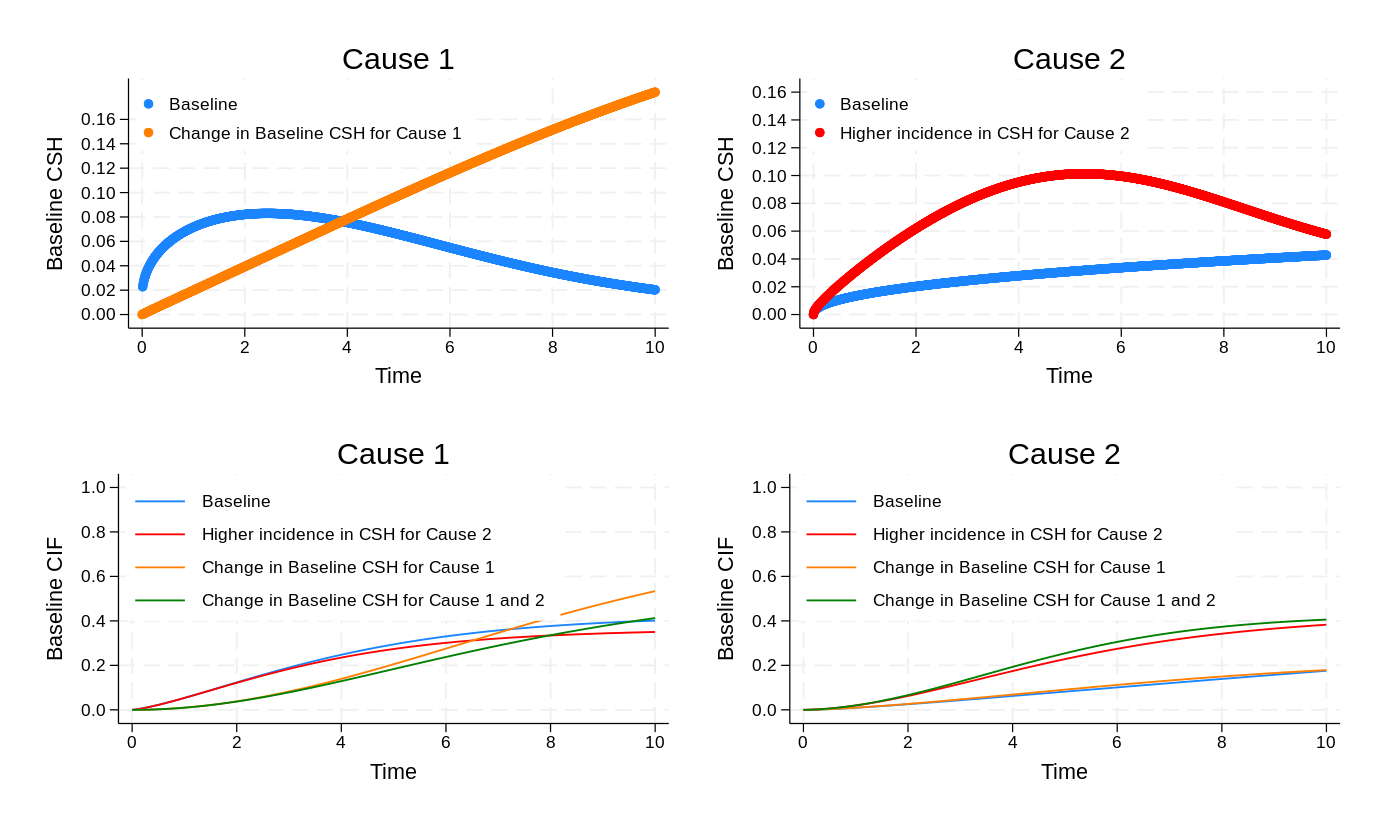


Figure A2: Simulated baseline cause-specific hazards and cumulative incidence functions where all variables have baseline values of 0 with an additional independent validation dataset with a different baseline hazard for cause 1 (orange) and a higher incidence of cause 2 (red).

Using the all-cause hazards derived from both events i.e. $h\left( t \right)= h_{1}^{cs}\left( t \right)+h_{2}^{cs}\left( t \right)$, the survival time for all-causes was simulated for each individual $t_{i}^{sim}$. The event types were then chosen based on a binomial experiment, where the probability that the survival time for event 1 is,

$$\frac{h_{1}^{cs}\left( t_{i}^{sim} \right)}{h(t_{i}^{sim})}$$

Finally, the distribution of censoring times were simulated from an exponential distribution with $\lambda=0.05$. Follow-up was restricted to 10 years and any simulated times greater than were censored at 10 years

Stata code for the simulation is provided in the cov_effects.do and master_sim_data.do files located within the GitHub repository available at https://github.com/sarwarislam/cal-competing-risks.

1. Crowther MJ, Lambert PC. Simulating biologically plausible complex survival data. Statistics in Medicine. 2013 Apr;32:4118–34.

# Appendix B: Demonstration of Proposed Method for Internal Validation

Below we extend the simulation to also demonstrate the application of proposed methods for internal validation. This can be used in addition to the standard model checking techniques for model development. The first scenario below evaluates a fitted model with a mis-specified functional form, where the non-linear function for $x_{11}$ is not specified. In the second, we evaluate a model where the covariate $x_{14}$ is excluded from the model.

#### Mis-specified functional form for prediction model in derivation data

In Figure B1, where we have assumed linearity on $x_{11}$, by inspecting the difference in IPA, it indicates that there is mis-calibration for both cause-specific absolute risks. This leads to a slight mis-calibration of the all-cause absolute risks. Even though the curves are still very close to the 45-degree line, in practice, this may look a lot worse, in which case, this will be clear when comparing different models. One can probably determine a missing functional form in a variable from the calibration plot for cause 1, due to some mis-calibration for cause 2, it is unclear whether there is mis-specification in one of, or both of the models. To suitably identify the model on which mis-specification has occurred, we evaluate mis-calibration on the cause-specific model predictions shown in the bottom row in Figure B1. By investigating the curves and the IPA the cause-specific model predictions, it is clear that these are unchanged for cause 2 compared to Figure 1. However, the cause-specific model predictions for cause 1 show a reduction in IPA (from 27.39% in Figure 1 to 20.82%) due to the omission of the correct functional form for $x_{11}$, and we can also observe a deviation from the reference line for “perfect” calibration. Note that, in practice, we do not have the IPA for a “correctly” fitted model to compare against. However, the IPA measure, along with an AIC/BIC metric, may still be used in a similar way to aid in model selection during model development.


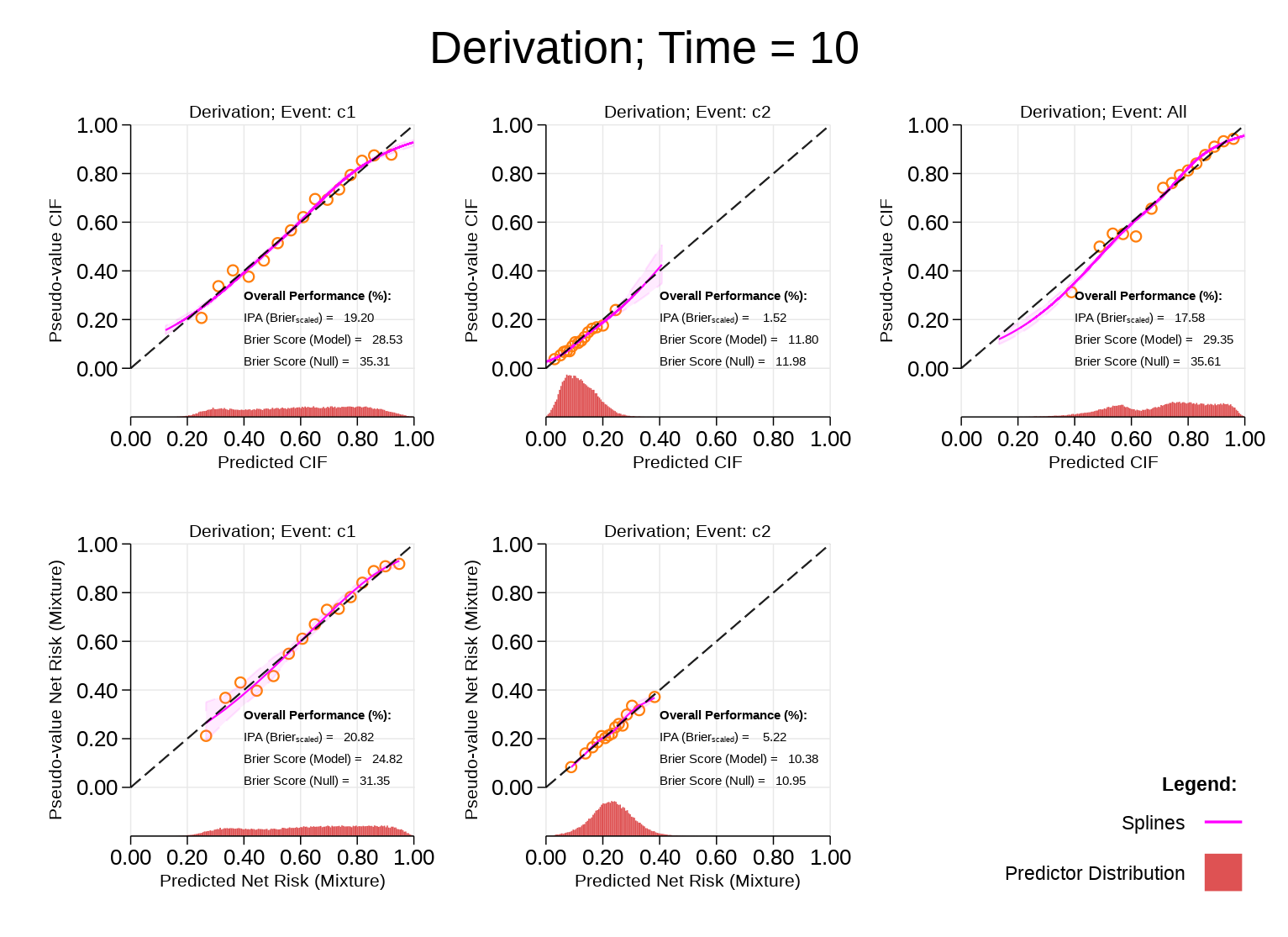


Figure B1: Calibration plots on the derivation data for predictions obtained from a correctly specified model for cause 2, and a mis-specified model with the incorrect functional form for $x_{1}$ for cause 1 at time T = 10. The cumulative incidence function (CIF) refers to the absolute risks.

#### Missing variable for prediction model in derivation data

Figure B2 show the calibration plots when a key categorical variable (variable $x_{14}$) has been omitted from the cause 1 model. Although only the cause 1 model excludes $x_{14}$, calibration on both cause-specific absolute risks are impacted. This can be mostly clearly seen by comparing the performance metrics between Figure 1 and Figure B2. However, by evaluating calibration on the cause-specific model predictions, we can identify which cause-specific model can be improved provided that a variable (i.e. $x_{k4}$) is available in the data during model development. Identifying a non-optimal model when a key variable is omitted is not always clearly identifiable from the calibration curves, as also demonstrated by Gerds et. al (2014) and as evident in Figure B2. However, as shown in the previous scenario, the IPA measure can still be used to identify a non-optimal prediction model. In this case, we again observe a drop in IPA for the cause-specific model predictions for cause 1 from 27.39% (for the DGM, Figure 1) to 12.28% for cause 1.


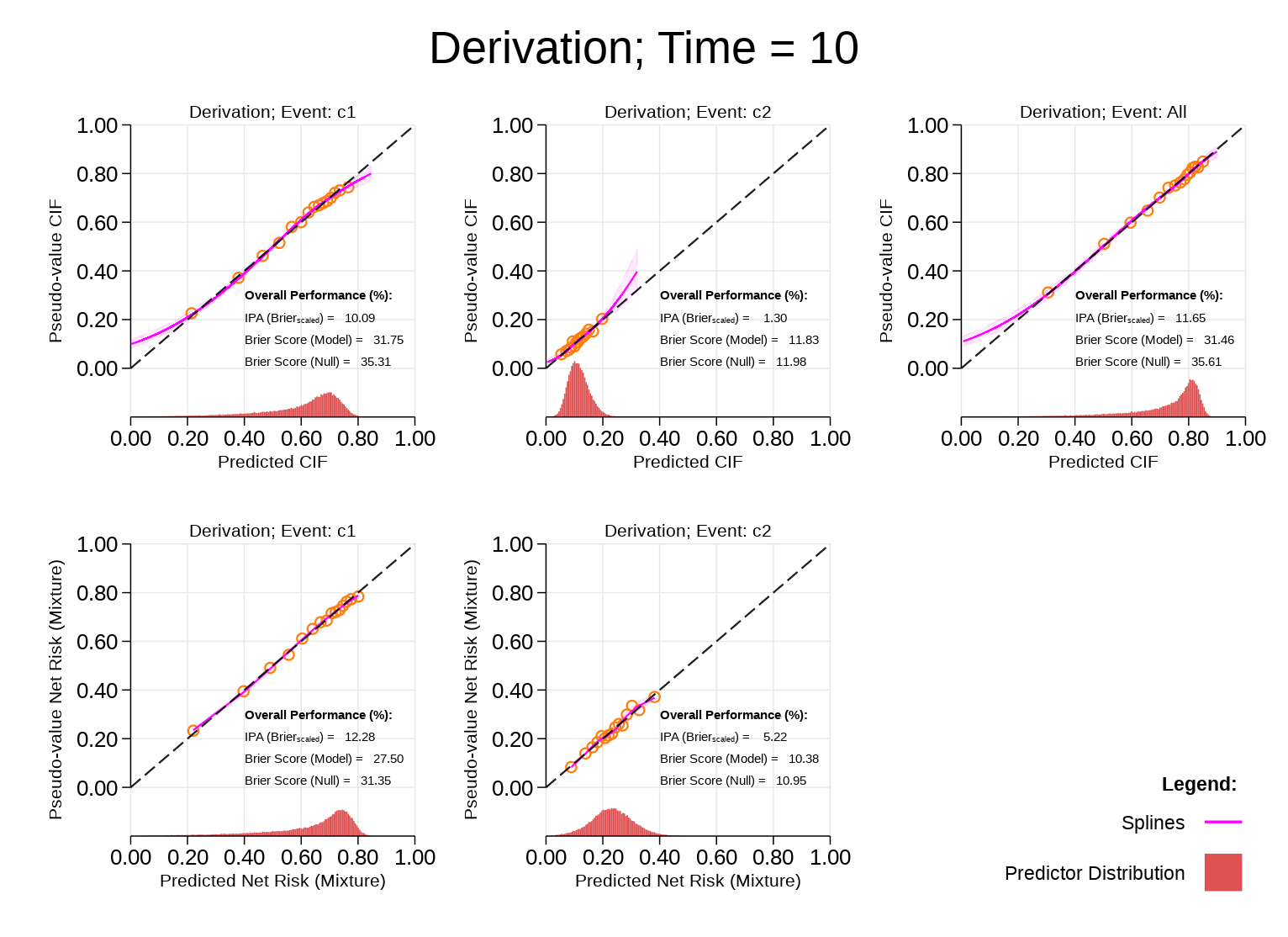


Figure B2: Calibration plots on the derivation data for predictions obtained from a correctly specified model for cause 2, and a mis-specified model with a variable $x_{4}$ not included for cause 1 at time T = 10. The cumulative incidence function (CIF) refers to the absolute risks.
